# Distribution of Visual and Oculomotor Alterations in a Clinical Population of Children with and without Neurodevelopmental Disorders

**DOI:** 10.3390/brainsci11030351

**Published:** 2021-03-10

**Authors:** Carmen Bilbao, David Pablo Piñero

**Affiliations:** 1 Department of Optometry, Policlínica Alto Aragón, 22003 Huesca, Spain; carmenbill0@gmail.com; 2 Group of Optics and Visual Perception, Department of Optics, Pharmacology and Anatomy, University of Alicante, 03690 Alicante, Spain; 3 Department of Ophthalmology, Vithas Medimar International Hospital, 03016 Alicante, Spain

**Keywords:** neurodevelopmental disorders, dyslexia, attention deficit/hyperactivity disorder, developmental coordination disorder, accommodation, binocular vision, stereopsis, oculomotricity, DEM, convergence

## Abstract

A prospective, non-randomized comparative study was conducted to compare the distribution of oculomotor and visual alterations in children with neurodevelopmental disorders and healthy children without such disorders. Sixty-nine children (aged 6–13 years) were enrolled and divided into three groups: a control group (CG) of 23 healthy children; a group of 18 healthy children with oculomotor abnormalities (OAG); and a group of 28 children with a neurodevelopmental disorder (NDDG), with 15 cases of dyslexia, 7 cases of developmental coordination disorder (DCD) and 6 cases of attention deficit/hyperactivity disorder (ADHD). Significantly worse near stereopsis was found in NDDG compared with CG (*p* < 0.001) and OAG (*p* = 0.001). Likewise, a significantly lower amplitude of accommodation was found in NDDG compared with CG in both the right (*p* = 0.001) and left eyes (*p* < 0.001). No statistically significant differences between groups were found in the measurement of near and distance phoria (*p* ≥ 0.557), near point of convergence (*p* = 0.700) and fusional vergences (*p* ≥ 0.059). Significantly impaired oculomotor test scores were found in NDDG compared with CG (*p* < 0.001), with no significant differences between OAG and NDDG (*p* ≥ 0.063). The comparison between the three types of neurodevelopmental disorders included revealed the presence of a significantly lower amplitude of accommodation in children with DCD compared with dyslexics. Furthermore, less exophoria at near was present in children with dyslexia compared with children with ADHD (*p* = 0.018) and DCD (*p* = 0.054). In conclusion, children with dyslexia, ADHD and DCD show an altered oculomotor pattern and a more reduced amplitude of accommodation, not always compatible with the diagnostic criteria of an accommodative insufficiency. Accommodative and binocular vision problems are not always present in these children and cannot be considered an etiologic factor.

## 1. Introduction

Neurodevelopmental disorders are characterized by early-onset deficits of variable severity in personal, social, academic or occupational functioning, as defined in the Diagnostic and Statistical Manual of Mental Disorders 5th Edition (DSM-5) [1]. Different conditions are included in this group of disorders, such as intellectual disability, autism spectrum disorder, specific learning disorders (including dyslexia), attention deficit/hyperactivity disorder (ADHD), motor disorders (including developmental coordination disorder, DCD) and communication disorders [1]. The onset of all these disorders is in the developmental period. The range of developmental deficits may vary from very specific limitations of learning or control of executive functions to global impairments of social skills or intelligence [2]. Individuals with dyslexia are characterized by specific and persistent reading problems [1,3], whereas children with ADHD present impairing levels of inattention, disorganization and/or hyperactivity-impulsivity [1]. Among neurodevelopmental motor disorders, DCD is characterized by deficits in the acquisition and execution of coordinated motor skills, and is manifested by clumsiness and slowness or inaccuracy in the performance of motor skills that cause interference with activities of daily living [1]. All these neurodevelopmental conditions have been associated with different alterations of ocular movements with potential impact on learning and reading activities [4,5,6,7,8], leading in some cases to the establishment of incorrect causal relationships [9], even though reading difficulties are not relevant for the diagnostic criteria of DCD or ADHD [1]. Furthermore, there are even doubts about the most adequate criteria for diagnosing oculomotor alterations in this type of disorders [9].

Besides oculomotricity, other authors have reported a large variety of visual problems associated with children with neurodevelopmental disorders, which may have also promoted the idea of a visual origin for this type of disorders [10,11,12,13]. The most frequent are refractive error problems, and oculomotor, accommodative and binocular problems [10,11,12,13]. However, these investigations only suggest the potential for visual problems to be a comorbidity in these conditions, and do not indicate that these alterations are causal factors and can be used as a diagnostic criterion for neurodevelopmental disorders, whose diagnosis is very complex and must be done under the criteria of the neuropediatrician and the educational psychologist. The hypothesis of dorsal stream vulnerability has been proposed as an explanation for a cluster of problems that are common to many neurodevelopmental disorders, including conditions such as poor motion sensitivity, visuomotor spatial integration for planning actions, attention and number skills [14,15]. This is being investigated as the source of some visuoperceptual difficulties that have been described in children with this type of disorders. The aim of the current study was to analyze, in a clinical population of a specialized center, the distribution of oculomotor and visual alterations defined according to standard clinical tests in children with three completely different types of neurodevelopmental disorders, and to compare the distribution with that obtained in healthy children without ocular pathology but with and without oculomotor anomalies.

## 2. Materials and Methods

### 2.1. Patients

A total of 69 children with ages ranging from 6 to 13 years old were enrolled in this prospective, non-randomized comparative study conducted at the Department of Optometry of the Policlínica Alto Aragón (Huesca, Spain). A complete explanation about the nature and aim of the study was provided to all parents of the enrolled children, who provided written informed consent allowing them to participate. The research adhered to the tenets of the Declaration of Helsinki and was revised and approved by the ethics committee of the University of Alicante (exp. UA-2018-02).

The recruitment of patients was performed in an Optometry Unit that was specialized in children’s vision. Children are normally referred by an ophthalmologist to this unit to undergo a complete visual performance examination, including analysis of accommodative, binocular and oculomotor functions. After explaining the nature of the study, if the parents of the children provided consent to their inclusion in it, a complete visual evaluation was performed, including an oculomotor evaluation with the Developmental Eye Movement (DEM) test. The result of this test was used to differentiate between children with and without oculomotor abnormalities. After this, each child was evaluated by a neuropediatrician to rule out the presence of any neurodevelopmental disorder. Besides this, all children diagnosed by the Child Development Unit of the clinic with a neurodevelopmental disorder are commonly referred to the Optometry Unit (after ophthalmological examination) to perform a complete visual performance evaluation. The parents of all these children with neurodevelopmental disorders were informed and asked about the potential participation of their children in the current study. Only data from those children whose parents gave written consent to be included in the study are analyzed in the current report.

Among children recruited for the study, three groups were clearly differentiated:

Control group (CG): 23 children with a mean age of 9 years old (median 9.0 years). The inclusion criteria for this group were healthy, ametropic (corrected with spectacles) or emmetropic children aged 6 to 13 years who achieved a corrected distance visual acuity (CDVA) of 0.00 logMAR (20/20 Snellen) or better. Exclusion criteria were any ocular or systemic disease active at the time of examination, previous visual training, as well as any previous ocular surgery. This group was examined by the neuropediatrician to rule out the presence of any neurodevelopmental disorder.

Group of healthy children with oculomotor abnormalities (OAG): 18 children with a mean age of 7.9 years old (median 8.0 years). The inclusion criteria for this group were children aged between 6 and 13 years, who underwent refractive correction if necessary, for more than 6 months, with absence of manifest strabismus and presence of oculomotor abnormality detected with the Developmental Eye Movement (DEM) test according to its normative data [16]. Specifically, the criterion for children to be recruited into the OAG group was the presence of a type II or type IV oculomotor pattern according to the DEM normative data. This group was examined by the neuropediatrician to rule out the presence of any neurodevelopmental disorder. Exclusion criteria were also any ocular or systemic disease active at the time of examination, previous visual training, as well as any previous ocular surgery.

Group of children diagnosed with a neurodevelopmental disorder (NDDG): 18 children with a mean age of 8.7 years old (median 9.0 years). A speech therapist, psychologist and neuropediatrician evaluated the conditions of these children and made a consistent diagnosis of neurodevelopmental disorder according to DSM-5 diagnostic criteria [1,17]. Dyslexia was present in 15 children, ADHD in 7 children, and a diagnosis of the motor disorder DCD was made in the remaining 6 children. According to previous studies, these children were expected to have oculomotor abnormalities in most cases [4,5,6,7,8,9]. The same exclusion criteria as those used in the other two groups were considered. No comorbidity of neurodevelopmental disorders was present in any case.

### 2.2. Visual Examination

A complete visual examination was performed after an exhaustive ophthalmologic evaluation of ocular health, which included measurement of uncorrected distance visual acuity (UDVA) and corrected distance visual acuity (CDVA), and manifest and cycloplegic refraction. After this, the following battery of accommodative, binocular and oculomotor tests was performed in all children with their best correction:

- Cover-uncover test: as no cases of strabismus were included, this test was performed to detect and measure the heterophoria at distance and near vision (40 cm) under photopic conditions. The quantification of the deviation was performed with a prism bar.
- Maddox rod test: this test allows a fast measurement of the heterophoria and has been used in many studies [18].
- Near point of convergence (NPC): the point of rupture and recovery of the fusion was detected using the Lang bar. Patients were instructed to indicate when the figure of the Lang bar became double (break) during the approach of the bar towards the eye and when it became single afterwards while the figure was moved away from the eye (recovery). The measurement was repeated 3 times, and the mean of the three measurements was recorded [19].
- Stereopsis: this was evaluated at near under photopic conditions using the Titmus Wirt test (Precision Vision, Woodstock, IL, USA) and at distance by means of the software Smarthings4vision (SmarThings4Vision, Zaragoza, Spain). Although the Titmus Wirt is a stereotest with monocular cues [20], it has been shown to be valid for the evaluation of stereopsis in clinical studies [21]. The test used for measuring the distance stereopsis (6 m) consisted of the identification of the protruding circle from those presented on the screen until a situation in which the subject was not able to discriminate the image in 3D (4 flat figures).
- Four-dot Worth test: this was used to determine if there was unilateral suppression under binocular conditions [22].
- Fusional vergence measurement: the base-in (divergence) and base-out (convergence) fusional vergences at distance and near were characterized by means of the points of blurring (first detection of blurring), rupture (first detection of double vision) and recovery (recovery of single vision) using a prism bar. This task was carried out under photopic conditions using for distance (6 m) an optotype corresponding to VA = 0.7, and for near (40 cm) also the optotypes of the chart corresponding to VA = 0.7 [23].
- Monocular and binocular accommodative facility: this was evaluated using a lens flipper of ± 2.00 D. The patient was asked to indicate when the letters of an optotype equivalent to VA = 0.7 presented at 40 cm could be seen clearly with the positive lenses and afterwards with the negative lenses. One cycle was considered each time the optotype was seen clearly with a positive lens and afterwards with a negative lens. The number of cycles achieved in 1 min was recorded [24].
- Positive/negative relative accommodation: negative relative accommodation (NRA) was measured with a phoropter by adding positive lenses in 0.25-D steps while the child was fixating at an optotype at 40 cm until the perception of blurring or double vision. The same procedure was followed to measure the positive relative accommodation (PRA), but using negative lenses [24].
- Amplitude of accommodation: this was measured using negative lenses monocularly while the child maintained fixation at an optotype corresponding to VA = 0.7 at 40 cm under photopic conditions. This is a test that provides slightly higher values than those obtained by the Donders method [25].
- Monocular Estimation Method (MEM) test: this was used to evaluate the accommodative response at 40 cm under ambient light. For this purpose, the child was asked to fixate at an optotype placed at 40 cm while the examiner observed the retinoscopic reflex and neutralized it with spherical lenses [23].
- NSUCO test (Northeastern State University College of Optometry’s Oculomotor): this is a standardized procedure with scoring criteria to characterize the pursuit and saccadic eye movements [26]. With this test, a trained examiner subjectively evaluates eye and saccadic movements considering four performance areas for smooth pursuit and saccadic movements: ability, accuracy, head movement and body movement [27]. The patient was sitting in front of the examiner who conducted the test binocularly using small colored spheres of 0.5 cm in diameter mounted on a rod as fixation stimuli that were presented at 40 cm. The stimulus was moved circumferentially (20 cm in diameter approximately) clockwise and counterclockwise to evaluate the smooth pursuits while children were asked to alternate the fixation between two stimuli separated horizontally by 20 cm to evaluate the saccadic movements [26,27]. The scoring criteria used for this test were as follows:
  ○ Smooth pursuits:
    ▪ Patient’s ability to perform two rotations (ability):
      ➢ 1 point: half rotation not completed
      ➢ 2 points: half rotation
      ➢ 3 points: 1 rotation in each direction
      ➢ 4 points: 2 rotations in one direction
      ➢ 5 points: 2 complete rotations
    ▪ Patient’s ability to perform two rotations without refixations (accuracy):
      ➢ 1 point: more than 10 refixations
      ➢ 2 points: 5 to 10 refixations
      ➢ 3 points: 3 to 4 refixations
      ➢ 4 points: 2 refixations or less
      ➢ 5 points: no refixations
    ▪ Patient’s ability to perform two rotations without head or body movements:
      ➢ 1 point: exaggerated body or head movement
      ➢ 2 points: large or moderate movement
      ➢ 3 points: slight movements but constant
      ➢ 4 points: slight movements but intermittent
      ➢ 5 points: no head or body movements
  ○ Saccadic movements
    ▪ Patient’s ability to perform 5 cycles of change of fixation between the two stimuli presented (ability):
      ➢ 1 point: 1 cycle or no ability
      ➢ 2 points: 2 cycles
      ➢ 3 points: 3 cycles
      ➢ 4 points: 4 cycles
      ➢ 5 points: 5 cycles
    ▪ Patient’s ability to perform 5 cycles of change of fixation without correcting refixations (accuracy):
      ➢ 1 point: very significant hyper- or hypometric movements
      ➢ 2 points: large to moderate hyper- or hypometric movements
      ➢ 3 points: slight hyper- or hypometric movements but constant
      ➢ 4 points: slight hyper- or hypometric movements but intermittent
      ➢ 5 points: no correcting refixations
    ▪ Patient’s ability to perform 5 cycles of change of fixation without head or body movements: same scoring as for smooth pursuits.
- DEM test (Developmental Eye Movement): this is a validated test to evaluate oculomotricity during reading in children from 6 to 14 years old [28]. The test consisted of observation while the child was asked to read four different sheets containing numbers:
  ○ Demo sheet: it is used to check the ability of the patient to read numbers and if the test could be performed.
  ○ Sheets A and B: they contained two columns of numbers listed vertically that the child was asked to read without the help of the finger as an indicator.
  ○ Sheet C: sheet containing letters displayed in horizontal lines.

The time needed by the child to read sheets A, B and C was recorded as well as the number and type of mistakes. A ratio (Horizontal Time/Vertical Time) was calculated with this data, which is used as the main outcome of the test [28].

All measurements were performed by the same experienced examiner (CB). Likewise, the same clinician (CB) performed all the diagnoses of accommodative and non-strabismic binocular dysfunctions strictly following the criteria previously defined in the peer-reviewed literature, which are summarized in Table 1 and Table 2.

### 2.3. Statistical Analysis

The statistical data analysis was performed using the software SPSS version 15.0 for Windows (IBM, Armonk, NY, USA). The Kolmogorov–Smirnov test confirmed that most samples did not follow a normal distribution and therefore non-parametric tests were used, considering a statistical correction for multiple comparisons. The Kruskal–Wallis test was used to analyze the significance of differences in a great variety of clinical variables between the groups involved in the study, with a post-hoc analysis performed using the Mann–Whitney test adjusted with the Bonferroni correction. The chi-square test was used to assess the statistical significance of differences between groups for percentages. All statistical tests were 2-tailed, and *p*-values less than 0.05 were considered statistically significant.

## 3. Results

### 3.1. Analysis of the Whole Sample

A total of 69 children (33 girls and 36 boys) with ages ranging from 6 to 13 years old were enrolled and were divided into three groups: CG (23 children), OAG (18 children) and NDDG (28 children), with no significant differences between groups in gender distribution (*p* = 0.213). Table 3 summarizes the main characteristics of these three groups and indicates which parameters showed statistically significant differences between groups.

Table 4 and Table 5 summarize the results of the accommodative and binocular examination for the three groups of children evaluated, respectively, indicating which parameters showed statistically significant differences between groups. For monocular accommodative facility, a difference between groups within the limit of statistical significance was found for the measurement of LE (*p* = 0.047), with a trend towards obtaining lower values in OAG and NDDG compared with CG.

Table 6 summarizes the results of the oculomotor examination for the three groups of children evaluated and indicates which parameters showed statistically significant differences between groups. As shown, statistically significant differences between groups were found in all the oculomotor variables evaluated with the NSUCO and DEM tests.

Regarding the diagnosis of accommodative and binocular anomalies, the results are summarized in Table 7. Only 2 non-strabismic binocular anomalies (convergence insufficiency and excess) and 2 accommodative alterations (accommodative excess and insufficiency) were detected in the sample evaluated.

After all this analysis, an additional comparison of healthy subjects (CG + OAG) with the group of subjects with neurodevelopmental disorders (NDDG) was also performed in order to confirm if the same significant differences were found. Specifically, significant differences between CG + OAG and NDDG were also found in distance (*p* = 0.009) and near stereopsis (*p* < 0.001), AA RE (*p* = 0.001), AA LE (*p* = 0.002), NSUCO scores (*p* ≤ 0.004), time required to read sheets A (*p* = 0.044), B (*p* = 0.047) and C (*p* = 0.007) of the DEM test, DEM ratio (*p* = 0.022) and number of errors in the DEM test (*p* = 0.018).

### 3.2. Detailed Analysis of the Neurodevelopmental Disorder Group (NDDG)

**Figure 1:**
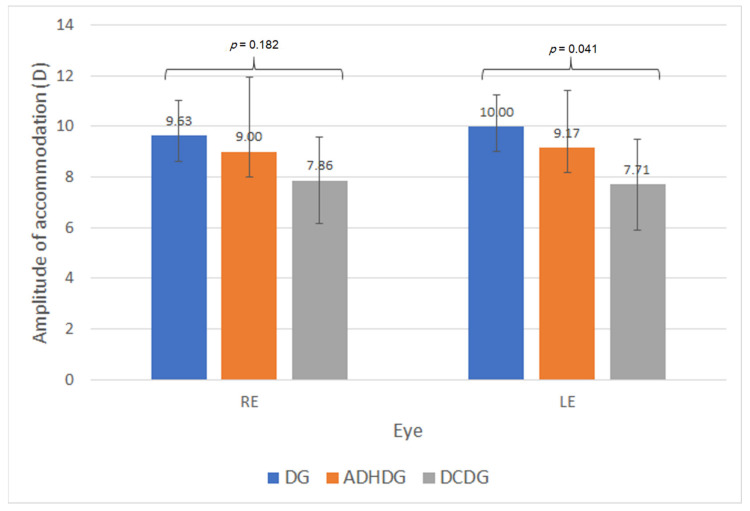
Mean amplitude of accommodation of the right (RE) and left eye (LE) in the subgroups of dyslexia (DG), attention deficit/hyperactivity disorder (ADHDG) and developmental coordination disorder (DCDG).

An additional analysis was performed in NDDG, performing a comparison between the different disorders included. Only four children (14.3%) from this group wore spectacles for the correction of their refractive error. Although the subgroups of dyslexia (DG), attention deficit/hyperactivity disorder (ADHDG) and developmental coordination disorder (DCDG) were small, a comparative analysis was performed to detect potential trends to be investigated further in future studies. No significant differences between DG, ADHDG and DCDG were found in refractive and visual acuity parameters (*p* ≥ 0.051). Concerning accommodative parameters, no significant differences were found in any of them (*p* ≥ 0.099), except for the amplitude of accommodation of LE (*p* = 0.041) (Figure 1). Specifically, the amplitude of accommodation was significantly lower in DCDG compared with DG (*p* = 0.007).

**Figure 2:**
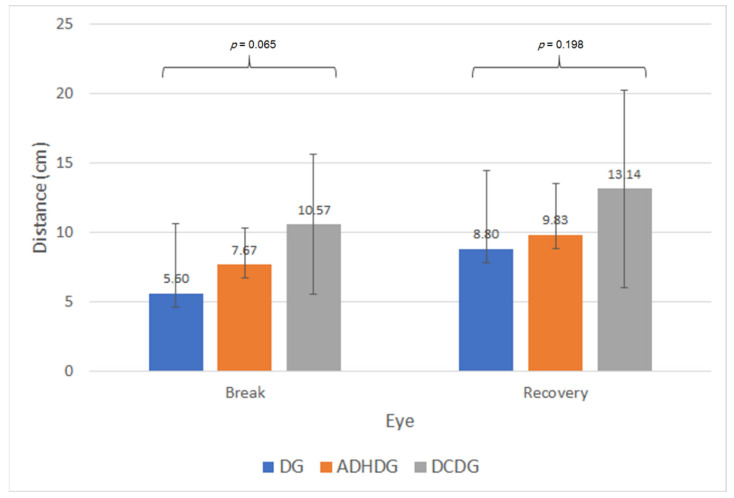
Mean values for the break and recovery points of the near point of convergence (NPC) in the subgroups of dyslexia (DG), attention deficit/hyperactivity disorder (ADHDG) and developmental coordination disorder (DCDG).

**Figure 3:**
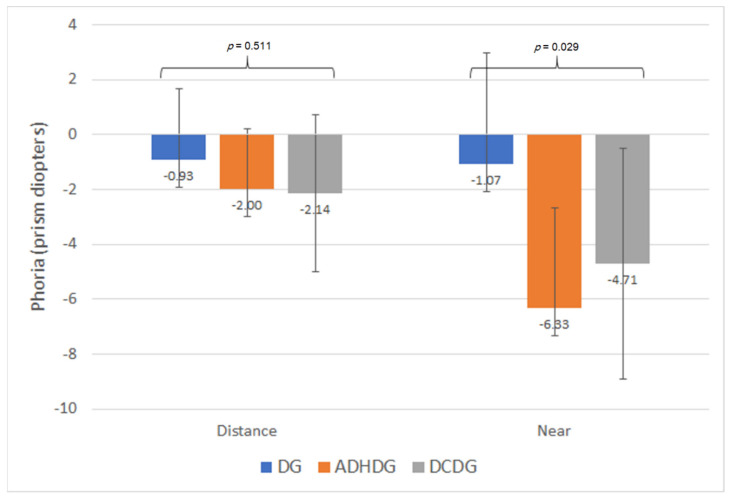
Mean values for the measurement with the cover test of the distance and near phoria in the subgroups of dyslexia (DG), attention deficit/hyperactivity disorder (ADHDG) and developmental coordination disorder (DCDG).

No statistically significant differences were found between learning disorder subgroups in NPC (break *p* = 0.065; recovery *p* = 0.198), although there was a trend towards obtaining closer break points for the NPC in DG compared with DCDG (Figure 2). No significant differences between subgroups were found in the measurement of the phoria at distance with the cover test (*p* = 0.511) and Maddox rod (*p* = 0.472). However, differences between subgroups in near phoria were detected when measured with the cover test (*p* = 0.029), but not when measured with the Maddox rod (*p* = 0.365), although the trend was the same (Figure 3). Specifically, more exophoria at near was present in ADHDG compared with DG (*p* = 0.018) (Figure 3). Likewise, a trend towards more exophoria in DCDG compared with DG was found, within the limit of statistical significance (*p* = 0.054) (Figure 3). Regarding fusional vergence parameters, no statistically significant differences were found in any of them between subgroups (*p* ≥ 0.156). In addition, no significant differences between DG, ADHDG and DCDG were found in distance (*p* = 0.609) and near stereopsis (*p* = 0.759). Concerning oculomotricity, no statistically significant differences between DG, ADHDG and DCDG were found in neither the NSUCO scores (*p* ≥ 0.113), DEM times (*p* ≥ 0.678) or DEM ratio (*p* = 0.403).

Table 8 shows the distribution of the diagnosis of accommodative and binocular anomalies in the three subgroups of children with neurodevelopmental disorders. There were no significant differences between subgroups of neurodevelopmental disorders in the percentage of cases with the diagnosis of accommodative and binocular anomalies.

## 4. Discussion

A great variety of scientific studies have confirmed the presence of significant alterations in oculomotricity in children with neurodevelopmental disorders [4,5,6,7,31,32,33,34,35]. However, these altered oculomotor patterns are not specific to this type of disorders [4], as they are also present in healthy children, and they have the potential to affect reading [36]. Therefore, children with oculomotor alterations and reading problems cannot be diagnosed as having a neurodevelopmental disorder, such as dyslexia, as the diagnosis of this type of conditions is very complex and requires a multidisciplinary approach [17]. In the specific case of dyslexia, it is mainly a linguistic, neurobiological disorder that directly affects reading, with the presence of associated visual alterations that are not an etiologic factor of the condition [37]. However, there are professionals still providing controversial visual therapies to treat dyslexia as well as other neurodevelopmental conditions, including color filter therapies, which have been uniquely shown to be useful in cases in which visual stress is also present [38]. In the current series, oculomotor alterations were detected in children with dyslexia, ADHD and DCD using two clinical tests not requiring advanced technology. Specifically, the NSUCO test was used to evaluate the ability, precision and associated head or body movements when performing smooth pursuits and saccades. This test has been shown to be useful to detect oculomotor alterations in children with sensory processing disorders [39]. Besides this tool, the DEM test was also used, which has been validated to detect and classify oculomotor anomalies, providing scores with high intra-subject test-retest reliability when the test is administered in an office setting and allowing a consistent classification of patients as pass or fail [28,40]. However, the only use of the DEM test for performing a diagnosis of an oculomotor anomaly was suggested to be potentially a source of error [41], and for this reason it was combined with the NSUCO test. Moiroud et al. [42] found in a comparative study using the DEM test that children with dyslexia took longer to read sheet C of the test than non-dyslexic children of similar chronological age. In the current series, children with neurodevelopmental disorders, namely, dyslexia, ADHD and DCD, showed longer times to read the three sheets of the DEM test as well as higher DEM ratio and number of errors. Raghuram et al. [32] also found impaired scores in the DEM test in children with dyslexia. Furthermore, no significant differences in NSUCO and DEM outcomes were found between neurodevelopmental disorder subgroups (dyslexia, ADHD and DCD) suggesting the presence of an oculomotor alteration in the three subgroups evaluated.

The results of the current study in terms of oculomotricity are consistent with those reported by previous authors [4,5,6,7,31,32,33,34,35]. Mahone et al. [5] found deficits in oculomotor response (response inhibition) in 60 children with ADHD compared with 60 controls. Bucci et al. [7] confirmed that dyslexic children had worse binocular coordination during and after the saccade, without showing the stereotyped pattern of disconjugacy (divergence during the saccade and convergence after the saccade). Sumner et al. [33] found that deficits in maintaining engagement in fixation and pursuit tasks with more anti-saccade errors were present in a group of children with DCD compared with a group of controls. An improvement of 75–100% in visual pursuit, fixation, ocular alignment and convergence was reported in children with DCD performing an 18-week visual therapy program [43]. However, in the current series, significant oculomotor alterations were also found in a sample of children without neurodevelopmental disorders, confirming that this type of alterations are not only present in neurodevelopmental disorders and cannot be an etiologic factor of these complex disorders. Indeed, no significant differences were found in NSUCO and DEM tests between children with oculomotor abnormalities but without neurodevelopmental disorders and those children with a clear diagnosis of a neurodevelopmental disorder. Palomo-Álvarez and Puell [36] demonstrated that poor horizontal scanning characterized by means of the DEM test was present in poor readers, suggesting that oculomotor alterations could be a factor slowing the reading speed without the presence of neurodevelopmental disorders. In any case, the oculomotor dysfunction that is present in the three neurodevelopmental disorders evaluated may have a relevant impact on some difficulties of ADHD children, such as visual search and visual attention abilities [44], as well as on visuomotor integration abnormalities of children with DCD [33,45]. The magnitude and extent of this impact should be investigated further in future studies.

Besides oculomotor alterations, some studies have reported a relatively high prevalence of accommodative and binocular anomalies in children with specific learning disorders [8,10,14,32,46]. As happened with oculomotor deficits, accommodative and binocular alterations have also been suggested to be a potential etiologic factor in some neurodevelopmental disorders. In the current comparative study, a significantly lower amplitude of accommodation in RE and LE was found in the group of children with neurodevelopmental disorders compared with the control group, which was a clinical population of children asking for a routine visual examination. This result is consistent with those reported by Feizabadi et al. [14], who reported significantly higher values of the near point of accommodation in a group of dyslexic children compared with 40 controls. Likewise, Raghuram et al. [31] found that 55% of a sample of children with dyslexia presented accommodative deficits compared with 9% of a group of controls. Muzaliha et al. [46] found in a large sample of children with learning disability that 28.3% of them had poor accommodative amplitude. According to strict scientific diagnostic criteria [23,29,30], a somewhat higher percentage of cases with accommodative excess (14.3% vs. 13.0%) and insufficiency (17.9% vs. 8.7%) was present in the group of children with neurodevelopmental disorders compared with controls, but these differences did not reach statistical significance. This confirms that although there is a trend towards a less accurate accommodative response in children with neurodevelopmental disorders, an accommodative dysfunction is not always present in these cases. Therefore, an accommodative cause for these specific disorders is completely impossible and should not be considered anymore. In the current sample, cases of accommodative infacility were not found following the strict diagnostic criteria defined, although a significantly lower monocular accommodative facility was found in LE in NDDG compared with CG. However, Hussaindeen et al. [10] reported that 67% of children with learning disability presented this condition, and Muzaliha et al. [46] reported a percentage of 26.0%. Differences in the diagnostic criteria used for accommodative infacility and even for learning or neurodevelopmental disorder seem to be one of the main reasons for this discrepancy.

No significant differences between CG, OAG and NDDG were found in NPC break and recovery in the current series. This is consistent with the results of Feizabadi et al. [14]. Likewise, no significant differences in the measurement of distance and near phoria nor in the measurement of fusional vergences were found in the present study between CG, OAG and NDDG. This contrasts with the results of other authors reporting limitations in fusional vergences [8,10,32,43]. Kapoula et al. [8] reported that a more remote NPC and a significantly reduced divergence at both near and distance were present in 57 dyslexic children compared with a sample of 46 non-dyslexic children. Muzaliha et al. [46] found that 12.1%, 45.7%, 37.4% and 66.3% of cases from a sample of 1010 children with learning disability evaluated had a poor convergence break, poor convergence recovery, poor divergence break and poor divergence recovery, respectively. Likewise, Raghuram et al. [32], as in our study, did not find a significant difference in the percentage of cases with vergence deficits comparing children with and without dyslexia. Differences in the definition of poor vergence response or vergence deficit may account for the discrepancies between studies. For this reason, future studies should be conducted following standard procedures and definitions of accommodative and binocular disorders to minimize the high variability among studies evaluating the prevalence of this type of disorders in children with dyslexia, ADHD or DCD. In the current sample, no significant differences were found in the percentage of cases with convergence insufficiency (26.1% vs. 25.0%) and excess (0% vs. 3.6%) between control and neurodevelopmental disorder groups. The percentage of cases with convergence insufficiency in our series is in the magnitude of those reported by other authors in samples of children with neurodevelopmental disorders [10,44]. It should be noted here that the control group was composed of children attending for a visual examination to the optometry department of a clinic after a comprehensive ophthalmologic examination. This may be considered a potential source of bias, as some parents visit vision specialists when there are complaints or signs suggesting a potential problem.

Finally, a comparison between neurodevelopmental disorder subgroups was performed, although the samples were reduced for performing such comparisons. Indeed, the outcomes obtained should be considered as trends to be confirmed in future studies. To our knowledge, this is the first comparison in terms of visual skills performed between children with dyslexia, ADHD and DCD. Differences between neurodevelopmental disorders were found in terms of amplitude of accommodation, with lower values for children with DCD compared with those with dyslexia. These differences only reached statistical significance for the measurement in the LE, possibly due to the limitation in the sample size, as the same trend was observed in the RE. Concerning the NPC, a trend towards measuring closer break points was found in dyslexics compared with DCD children, with a difference close to the limit of statistical significance. Significantly more exophoria was present in children with ADHD compared with dyslexics, and a trend within the limit of statistical significance towards more exophoria in children with DCD compared with dyslexics was also found. Therefore, some differences in terms of accommodative response and ocular alignment at near seem to be present between children with dyslexia, ADHD and DCD. This may explain differences between studies evaluating the visual skills of children with neurodevelopmental disorders, including different populations of dyslexics and children with ADHD and DCD. These differences may be attributable to the differences between these three conditions in terms of their pathogenesis and impact on brain activity. More research is still needed on these issues to allow clinicians to better understand these conditions.

This study has several limitations that should be acknowledged. First, the subjectivity of the oculomotor tests can be considered a limitation, as discussed in a previous study by our research group [4]. For this reason, two tests were used that can be easily used in clinical practice. Likewise, all oculomotor tests were performed in the current study by the same experienced examiner to avoid potential inter-observer variability, which is normally present when performing clinical tests. In any case, although videoculography is considered the most adequate tool for characterizing oculomotor anomalies due to the objectivity of this type of examination, there is still a need for standardized diagnostic criteria using this advanced technology [9]. The specific use of the DEM test to evaluate oculomotricity can also be considered a limitation, as there is some controversy about what it is really measuring, with some authors suggesting that the DEM test outcome is not exactly correlated with saccadic eye movement skills, but is more related to reading performance and visual processing speed [47]. For this reason, the NSUCO test was also used to confirm that the oculomotor response was altered. Significantly reduced near and distance stereopsis was also present in the group of children with neurodevelopmental disorders compared with controls, which may be consistent with an altered DEM test outcome if it is correlated with visual processing, as reduced stereopsis is a sign of limited binocular visual processing. Besides this, there is also a limitation in the sample size of each subtype of neurodevelopmental disorders, but it should be considered that it is difficult to recruit children with a scientifically precise diagnosis of these conditions (dyslexia, ADHD or DCD) with no previous treatments or therapies. It should be considered that a great proportion of children with learning disorders can combine symptoms of dyslexia, ADHD and DCD [48]. Finally, it should be remarked that a large percentage of children without neurodevelopmental disorders but with oculomotor abnormalities was found in the current study. The main reason that may explain this curious finding is the specific characteristics of the clinic in which this study was developed. It should be considered that our clinic is specialized in children’s vision, and possibly several parents attended a consultation to find a solution for their child’s visual problem or to rule out whether their child’s academic problems were potentially related to visual problems. For this reason, the percentage of visual problems found in the clinical population evaluated cannot be extrapolated to the general population. However, the analysis of this clinical population is interesting because a complete characterization of different types of visual problems can be performed, including those that can be present in neurodevelopmental disorders.

## 5. Conclusions

Children with dyslexia, ADHD and DCD show an altered oculomotor pattern that is not specific to this population, since this type of alteration is also present in children without neurodevelopmental disorders. A more reduced range of accommodation is present in children with these neurodevelopmental disorders, especially DCD, although this reduction is not always compatible with the diagnostic criteria for accommodative insufficiency. In fact, the percentage of cases with accommodative insufficiency and excess in the group of children with neurodevelopmental disorders did not differ in the current study from those found in a healthy clinical population without such disorders. Furthermore, no significant differences were found in the measurement of NPC and distance and close phoria between children with and without neurodevelopmental disorders, with a trend towards a more reduced amplitude of accommodation and more remote NPC in children with DCD. Likewise, a trend towards more exophoria at near was found in children with ADHD, but not always compatible with the diagnosis of convergence insufficiency. Therefore, accommodative and binocular problems are not always present in children with neurodevelopmental disorders and cannot be considered an etiological factor for these complex disorders. More studies are still needed to characterize the real impact of these oculomotor, accommodative and binocular vision problems in children with neurodevelopmental disorders, examining possible neuropsychological insights for this population of patients and evaluating if abnormalities in visual behavior could be one of the early signs of neurodevelopmental disorders, as some authors suggest [49,50]. Furthermore, as oculomotor alterations are present in children with neurodevelopmental disorders, such as dyslexia, ADHD or DCD, children presenting any type of oculomotor anomaly in a routine visual examination should be revised to rule out the presence of this type of disorders.

## Figures and Tables

**Table 1:** Diagnostic criteria defined in the peer-reviewed literature for non-strabismic binocular anomalies that have been used in the current study.

Type of Anomaly	Author, Year	Clinical Signs
Convergence insufficiency (CI)	Garcia et al., 2002 [29]	Exophoria > 6Δ Cover Test (Near) NPC: >10/17 cm PFV: ≤11/14/3Δ, at least one of three responses of blur/break/recovery AC/A Calculated <3/1 BAF ≤3 cpm. Difficulty in clearing +2 D (±2.00 D) MEM: <+0.25 NRA: ≤−1.50 D
Convergence excess (CE)	Garcia et al., 2002 [29]	Esophoria > 2Δ Cover Test (Near) NFV ≤8/16/7Δ, at least one of three responses of blur/break/recovery AC/A > 7/1 BAF ≤3cpm. Difficulty in clearing −2D (±2.00 D) PRA ≤1.25 D
Divergence insufficiency (DI)	Dwyer, 1991 [23]	Esophoria at far = 3Δ NFV = 0 blur/break/recovery AC/A ≤3/1
Divergence excess (DE)	Dwyer, 1991 [23]	Exophoria > 5Δ (Far) PFV = 0 blur/break/recovery AC/A ≥ 6/1
Basic exophoria (BX)	Dwyer, 1991 [23]	Uncompensated exophoria at far and near Δ AC/A = 4.5/1
Basic esophoria (BE)	Dwyer, 1991 [23]	Uncompensated esophoria at far and near Δ AC/A = 4.5/1

Abbreviations: NPC, near point of convergence; PFV, positive fusional vergence; AC/A, convergence induced by accommodation per diopter of accommodation; BAF, binocular accommodative facility; MEM, monocular estimation method; NRA, negative relative accommodation; PRA, positive relative accommodation.

**Table 2:** Diagnostic criteria defined in the peer-reviewed literature for accommodative anomalies that have been used in the current study.

Type of Anomaly	Author, Year	Clinical Signs
Accommodative insufficiency (AI)	Cacho et al., 2002 [30]	AA at least 2D < minimum AA (age appropriate) of Hofstetter’s formula (15–0.25× age) Monocular push-up method MAF: ≤ 6 cpm. Difficulty in clearing −2 D (±2.00 D) BAF: ≤ 3 cpm. Difficulty in clearing −2 D (±2.00 D) MEM: >+0.75 D PRA: ≤1.25 D Symptoms in near vision
Accommodative excess (AE)	Garcia et al., 2002 [29]	MAF: ≤ 6 cpm. Difficulty in clearing +2 D (±2.00 D) BAF: ≤ 3 cpm. Difficulty clearing +2 D (±2.00 D) MEM: <+0.25 D NRA: ≤1.50 D Variable visual acuity Symptomatology in far vision
Accommodative infacility (AIN)	Dwyer, 1991 [23]	Disorder of facility

Abbreviations: MAF, monocular accommodative facility; BAF, binocular accommodative facility; AA, amplitude of accommodation; MEM, monocular estimation method; NRA, negative relative accommodation; PRA, positive relative accommodation.

**Table 3:** Summary of the main characteristics of the three groups evaluated in the study: CG, control group; OAG, group of children with oculomotor abnormalities; NDDG, group of children with neurodevelopmental disorders.

Mean (SD) Median (Range)	CG (23)	OAG (18)	NDDG (28)	*p*-Value
**Age (years)**	9.0 (1.62) 9.0 (6.0 to 11.0)	7.9 (1.7) 8.0 (6.0 to 11.0)	8.7 (2.5) 9.0 (6.0 to 13.0)	0.138
**Sphere RE (D)**	0.29 (1.05) 0.00 (0.00 to 5.00)	0.06 (0.24) 0.00 (0.00 to 1.00)	0.18 (0.87) 0.00 (−1.75 to 4.00)	0.579
**Cylinder RE (D)**	−0.16 (0.64) 0.00 (−3.00 to 0.00)	−0.03 (0.12) 0.00 (−0.50 to 0.00)	−0.29 (0.91) 0.00 (−4.00 to 0.00)	0.590
**Sphere LE (D)**	0.31 (1.08) 0.00 (0.00 to 5.00)	0.04 (0.18) 0.00 (0.00 to 0.75)	0.04 (1.17) 0.00 (−4.00 to 4.00)	0.783
**Cylinder LE (D)**	−0.15 (0.55) 0.00 (−2.50 to 0.00)	−0.01 (0.06) 0.00 (−0.25 to 0.00)	−0.20 (0.64) 0.00 (−3.00 to 0.00)	0.798
**LogMAR CDVA RE**	0.01 (0.02) 0.00 (0.00 to 0.10)	−0.04 (0.07) 0.00 (−0.20 to 0.05)	−0.05 (0.18) 0.00 (−0.08 to 0.85)	0.003 * CG-OAG 0.005 * CG-NDDG 0.620 OAG-NDDG 0.008 *
**LogMAR CDVA LE**	0.0 (0.03) 0.00 (0.00 to 0.10)	−0.04 (0.07) 0.00 (−0.20 to 0.05)	−0.02 (0.9) 0.00 (−0.09 to 0.50)	0.005 * CG-OAG 0.004 * CG-NDDG 0.411 OAG-NDDG 0.014 *
**Distance stereopsis (sec arc)**	47.7 (23.53) 40.00 (18.00 to 100.00)	55.18 (21.11) 52.50 (18.20 to 100.0)	63.82 (19.18) 66.50 (10.00 to 80.0)	0.024 * CG-OAG 0.353 CG-NDDG 0.009 * OAG-NDDG 0.090
**Near stereopsis (sec arc)**	30.65 (23.90) 20.00 (20.00 to 100.00)	30.83 (18.91) 26.00 (20.00 to 100.0)	60.11 (71.22) 40.00 (0.00 to 400.0)	<0.001 * CG-OAG 0.211 CG-NDDG < 0.001 * OAG-NDDG 0.001 *
**NPC break (cm)**	6.96 (5.98) 4.00 (0.00 to 20.00)	6.50 (3.91) 5.00 (2.00 to 15.00)	7.29 (4.95) 6.00 (0.00 to 20.00)	0.700
**NPC recovery (cm)**	10.35 (6.18) 8.00 (3.00 to 25.00)	9.33 (4.03) 8.00 (4.00 to 19.00)	10.11 (5.81) 9.50 (3.00 to 25.00)	0.999

Abbreviations: RE, right eye; LE, left eye; D, diopter; CDVA, corrected distance visual acuity; NPC, near point of convergence. The results of the cover test are expressed as negative in the presence of exophoria and positive in the presence of esophoria. * *p*-values representing statistically significant differences (*p*-value < 0.05).

**Table 4:** Summary of the results of the accommodative examination in the three groups of children evaluated: CG, control group; OAG, group of children with oculomotor abnormalities; NDDG, group of children with neurodevelopmental disorders.

Mean (SD) Median (Range)	CG (23)	OAG (18)	NDDG (28)	*p*-Value
**AA RE (D)**	11.35 (2.76) 12.00 (4.00 to 15.0)	10.41 (2.34) 10.00 (6.0 to 14.00)	9.03 (1.98) 9.00 (5.00 to 13.0)	0.003 * CG-OAG 0.205 CG-NDDG 0.001 * OAG-NDDG 0.052
**AA LE (D)**	11.80 (2.67) 13.00 (4.00 to 15.0)	10.06 (2.79) 10.00 (3.00 to 14.00)	9.22 (1.85) 9.50 (5.00 to 13.0)	0.001 * CG-OAG 0.039 * CG-NDDG < 0.001 * OAG-NDDG 0.192
**MEM RE (D)**	0.12 (0.88) 0.25 (−2.00 to 1.50)	0.33 (0.65) 0.50 (−1.00 to 1.00)	0.46 (0.60) 0.50 (−1.00 to 1.50)	0.251
**MEM LE (D)**	0.11 (0.85) 0.25 (−2.00 to 1.50)	0.34 (0.63) 0.50 (−1.00 to 1.25)	0.43 (0.60) 0.67 (−1.00 to 1.50)	0.270
**MAF RE (cpm)**	11.90 (4.85) 13.00 (3.00 to 20.00)	9.11 (5.12) 8.50 (0.00 to 20.00)	9.44 (4.49) 10.00 (0.00 to 20.00)	0.099
**MAF LE (cpm)**	12.48 (5.19) 13.00 (3.00 to 20.00)	9.56 (5.52) 9.00 (0.00 to 21.00)	9.26 (4.67) 9.00 (0.00 to 20.00)	0.047 *
**BAF (cpm)**	9.39 (4.68) 12.00 (0.00 to 15.00)	7.93 (5.06) 9.00 (0.00 to 15.00)	9.15 (7.11) 9.00 (0.00 to 27.00)	0.710
**NRA (D)**	1.47 (0.99) 1.50 (0.00 to 3.00)	1.68 (1.25) 1.00 (0.00 to 4.00)	1.96 (0.91) 2.50 (0.00 to 3.00)	0.417
**PRA (D)**	−2.00 (1.41) −2.00 (−4.00 to 0.00)	−1.93 (1.10) −2.00 (−3.00 to −0.50)	−1.74 (1.17) −1.88 (−3.00 to 0.00)	0.957

Abbreviations: SD, standard deviation; AA, amplitude of accommodation; RE, right eye; LE, left eye; MEM; monocular estimation method; MAF, monocular accommodative facility; BAF, binocular accommodative facility; cpm, cycles per minute; NRA, negative relative accommodation; PRA, positive relative accommodation. * *p*-values representing statistically significant differences (*p*-value < 0.05).

**Table 5:** Summary of the results of the binocular examination in the three groups of children evaluated: CG, control group; OAG, group of children with oculomotor abnormalities; NDDG, group of children with neurodevelopmental disorders.

Mean (SD) Median (Range)	CG (23)	OAG (18)	NDDG (28)	*p*-Value
**Distance cover test (** **Δ)**	−1.56 (2.87) 0.00 (−10.00 to 3.00)	−0.78 (3.30) 0.00 (−8.00 to 8.00)	−1.46 (2.56) 2.56 (−8.00 to 4.00)	0.644
**Near cover test (** **Δ)**	−3.00 (4.98) 0.00 (−16.00 to 6.00)	−1.94 (5.14) 0.00 (−10.00 to 10.00)	−3.11 (4.50) −1.50 (−10.00 to 6.00)	0.823
**Distance Maddox phoria (** **Δ)**	−1.87 (3.77) 0.00 (−12.00 to 5.00)	−1.22 (3.89) 0.00 (−14.00 to 2.00)	−0.85 (3.19) −0.85 (−10.00 to 6.00)	0.557
**Near Maddox phoria (** **Δ)**	−3.78 (6.44) 0.00 (−20.00 to 8.00)	−1.78 (5.36) 0.00 (−16.00 to 10.00)	−1.93 (5.47) −2.00 (−10.00 to 12.00)	0.691
**Distance break point NFV (** **Δ)**	6.96 (2.88) 6.00 (4.00 to 14.00)	6.33 (1.85) 6.00 (4.00 to 10.0)	5.26 (2.36) 6.00 (0.00 to 10.0)	0.151
**Distance recovery point NFV (** **Δ)**	4.78 (4.03) 4.00 (2.00 to 20.00)	4.00 (2.08) 4.00 (2.00 to 8.0)	3.48 (1.72) 4.00 (0.00 to 6.0)	0.801
**Distance blur point PFV (** **Δ)**	10.19 (4.64) 10.00 (2.00 to 20.00)	10.64 (4.20) 10.00 (4.00 to 20.0)	6.89 (5.10) 8.00 (0.00 to 18.0)	0.059
**Distance break point PFV (** **Δ)**	12.74 (5.31) 12.00 (4.00 to 25.00)	13.61 (4.96) 14.00 (6.00 to 25.00)	12.00 (6.37) 14.00 (0.00 to 30.00)	0.661
**Distance recovery point PFV (** **Δ)**	9.30 4.69) 10.00 (0.00 to 20.00)	10.33 (4.51) 11.00 (4.00 to 20.00)	9.15 (5.33) 10.00 (0.00 to 18.00)	0.801
**Near blur point NFV (** **Δ)**	11.08 (2.38) 10.00 8.00 to 14.00)	8.8 (3.91) 8.00 (4.00 to 16.0)	8.47 (3.78) 8.00 (0.00 to 14.0)	0.082
**Near break point NFV (** **Δ)**	12.70 (3.84) 14.00 (4.00 to 18.00)	12.22 (3.28) 13.00 (8.00 to 18.0)	11.86 (4.55) 13.00 (0.00 to 18.0)	0.681
**Near recovery point NFV (** **Δ)**	9.65 (3.23) 10.00 (4.00 to 16.00)	9.11 (3.51) 10.00 (2.00 to 14.0)	8.71 (3.93) 10.00 (2.00 to 18.00)	0.761
**Near blur point PFV (** **Δ)**	21.33 (10.1) 25.00 (0.00 to 35.00)	20.06 (9.70) 22.50 (6.00 to 35.00)	16.40 (8.34) 20.00 (0.00 to 25.0)	0.227
**Near break point PFV (** **Δ)**	25.65 (12.47) 30.00 (0.00 to 40.00)	25.11 (11.00) 27.50 (10.00 to 40.00)	21.61 (7.80) 20.00 (8.00 to 40.00)	0.241
**Near recovery point PFV (** **Δ)**	19.74 (10.17) 20.00 (0.00 to 35.00)	17.28 (10.31) 20.00 (2.00 to 30.00)	16.25 (6.68) 17.00 (4.00 to 30.00)	0.266

Abbreviations: SD, standard deviation; NFV, negative fusional vergence; PFV, positive fusional vergence.

**Table 6:** Summary of the results of the oculomotor examination in the three groups of children evaluated: CG, control group; OAG, group of children with oculomotor abnormalities; NDDG, group of children with neurodevelopmental disorders.

Mean (SD) Median (Range)	CG (23)	OAG (18)	NDDG (28)	*p*-Value
***NSUCO test: smooth pursuits***				
**Ability**	4.3 (0.9) 5.0 (2.0 to 5.0)	2.2 (1.3) 2.0 (1.0 to 5.0)	2.3 (1.3) 2.0 (1.0 to 5.0)	<0.001 * CG-OAG < 0.001 * CG-NDDG < 0.001 * OAG-NDDG 0.813
**Precision**	4.2 (0.9) 4.0 (2.0 to 5.0)	2.3 (1.2) 2.0 (1.0 to 5.0)	2.3 (1.3) 2.0 (1.0 to 5.0)	< 0.001 * CG-OAG < 0.001 * CG-NDDG < 0.001 * OAG-NDDG 0.981
**Head/body movement**	4.1 (1.1) 5.0 (2.0 to 5.0)	2.1 (1.2) 2.0 (1.0 to 5.0)	1.5 (0.7) 1.0 (1.0 to 3.0)	<0.001 * CG-OAG < 0.001 * CG-NDDG < 0.001* OAG-NDDG 0.063
***NSUCO test: saccades***				
**Ability**	4.3 (0.8) 4.0 (2.0 to 5.0)	2.3 (1.2) 2.0 (1.0 to 5.0)	2.2 (0.8) 2.0 (1.0 to 4.0)	<0.001 * CG-OAG < 0.001 * CG-NDDG < 0.001 * OAG-NDDG 0.943
**Precision**	4.3 (0.8) 5.0 (2.0 to 5.0)	2.2 (1.2) 2.0 (1.0 to 5.0)	2.4 (1.3) 2.0 (1.0 to 5.0)	<0.001 * CG-OAG < 0.001 * CG-NDDG < 0.001 * OAG-NDDG 0.500
**Head/body movement**	4.3 (1.1) 5.0 (2.0 to 5.0)	2.1 (1.2) 2.0 (1.0 to 5.0)	1.6 (0.7) 1.5 (1.0 to 3.0)	<0.001 * CG-OAG < 0.001 * CG-NDDG < 0.001 * OAG-NDDG 0.237
***DEM test***				
**Time sheet A (s)**	20.7 (6.0) 19.0 (14.0 to 38.0)	25.1 (7.1) 24.0 (17.0 to 43.0)	26.93 (8.9) 25.0 (11.3 to 47.0)	0.011 * CG-OAG 0.013 * CG-NDDG 0.008 * OAG-NDDG 0.565
**Time sheet B (s)**	21.74 (5.5) 20.00 (15.0 to 37.0)	26.7 (7.1) 25.5 (19.0 to 47.0)	28.5 (9.4) 26.0 (11.4 to 48.0)	0.008 * CG-OAG 0.008 * CG-NDDG 0.007 * OAG-NDDG 0.660
**Time sheet C**	56.5 (21.5) 46.0 (37.0 to 107.0)	106.6 (50.2) 78.5 (46.0 to 240.0)	119.7 (64.0) 99.5 (30.3 to 247.0)	<0.001 * CG-OAG < 0.001 * CG-NDDG < 0.001 * OAG-NDDG 0.753
**DEM ratio**	1.3 (0.3) 1.3 (1.0 to 2.0)	2.1 (1.0) 1.6 (1.0 to 4.7)	2.2 (1.2) 1.7 (1.0 to 5.1)	<0.001 * CG-OAG < 0.001 * CG-NDDG < 0.001 * OAG-NDDG 0.973
**Number of errors**	3.5 (5.1) 2.0 (0.0 to 20.0)	5.4 (6.1) 2.5 (0.0 to 20.0)	12.0 (13.2) 6.5 (0.0 to 45.0)	0.037 * CG-OAG 0.275 CG-NDDG 0.014 * OAG-NDDG 0.152

Abbreviations: SD, standard deviation. * *p*-values representing statistically significant differences (*p*-value < 0.05).

**Table 7:** Distribution of the different accommodative and binocular anomalies diagnosed in the three groups of children evaluated: CG, control group; OAG, group of children with oculomotor abnormalities; NDDG, group of children with neurodevelopmental disorders.

Condition	CG (23)	OAG (18)	NDDG (28)	*p*-Value
Convergence insufficiency	*n* = 6 26.1%	*n* = 3 16.7%	*n* = 7 25.0%	0.745
Convergence excess	*n* = 0 0.0%	*n* = 1 5.6%	*n* = 1 3.6%	0.553
Accommodative excess	*n* = 3 13.0%	*n* = 4 22.2%	*n* = 4 14.3%	0.694
Accommodative Insufficiency	*n* = 2 8.7%	*n* = 3 16.7%	*n* = 5 17.9%	0.471

**Table 8:** Distribution of the different accommodative and binocular anomalies diagnosed in the three subgroups of children with neurodevelopmental disorders evaluated: DG, dyslexia group; ADHDG, group of children with attention deficit/hyperactivity disorder; DCDG, group of children with developmental coordination disorder.

Condition	DG (15)	ADHDG (6)	DCDG (7)	*p*-Value
Convergence insufficiency	*n* = 2 13.3%	*n* = 3 50.0%	*n* = 2 28.6%	0.208
Convergence excess	*n* = 1 6.7%	*n* = 0 0.0%	*n* = 0 0.0%	0.638
Accommodative excess	*n* = 3 20.0%	*n* = 1 16.7%	*n* = 0 0.0%	0.451
Accommodative insufficiency	*n* = 4 26.7%	*n* = 2 33.3%	*n* = 3 42.9%	0.118

## Data Availability

Data available on request due to privacy/ethical restrictions.
